# The Takara SARS-CoV-2 direct PCR detection kit delivers reliable results with throat wash specimens

**DOI:** 10.1016/j.nmni.2022.101027

**Published:** 2022-09-05

**Authors:** M. Nitsch, T. Stahlhut, O. Schildgen, V. Schildgen

**Affiliations:** Institut für Pathologie, Kliniken der Stadt Köln gGmbH, Klinikum der Privaten Universität Witten/Herdecke, Ostmerheimer Str. 200, D-51109 Cologne, Germany

**Keywords:** COVID-19, extraction free PCR, medical staff, SARS-CoV-2

Dear Editor,

Actually, severe acute respiratory syndrome coronavirus 2 (SARS-CoV-2) detection by quantitative polymerase chain reaction (qPCR) still represents the diagnostic gold standard regarding the global SARS-CoV-2 pandemic. Nevertheless, qPCR diagnostic is a rather time-consuming process depending on nucleic acid preparation and PCR-protocol, respectively. As diagnoses are time-sensitive to reduce the risk of viral transmissions and to avoid quarantine based staff shortage, fast turnover times are a mandatory requirement. As the pandemic has shown that both human and technical resources may quickly become limited, molecular diagnostic methods reducing hands-on time and technical effort while keeping high quality standards become highly important by extending already established diagnostic tests for future epidemic waves.

Therefore, we performed a two stage pilot study with the novel *in vitro diagnostics* (IVD) labeled Takara SARS-CoV-2 Direct PCR detection assay in which RNA of heated specimen-containing pretreatment-mix is added directly into the real-time reverse transcription-PCR (rt-RT-PCR) reaction.

First, we analysed the technical sensitivity of the assay by using two reference samples named BP3 (BP = “Bezugs-Probe”) and BP4 obtained by INSTAND e.V. (INSTAND, Düsseldorf, Germany). These samples, adjusted to viral RNA loads of about 10ˆ7 (BP3) and 10ˆ6 (BP4) copies/ml, are based on cell culture supernatants derived from SARS-CoV-2 infected Vero-cells [1]. Dilution series were prepared in duplicate to determine the correlation between viral RNA load and crossing threshold (Ct)-value in the Takara assay (Fig. 1). The average N-gene Ct-value difference between the undiluted reference samples BP3 (Ct 24.54) and BP4 (Ct 27.87) is 3.33 compared to 3.44 ± 0.64 previously reported by INSTAND e.V [1]. Copies of the N-gene could be detected down to 500 copies/ml sample taking into account that the measuring range of 10^ˆ^2 – 10^ˆ^3 copies/ml represents the detection limit in the given test system.

**Fig. 1:**
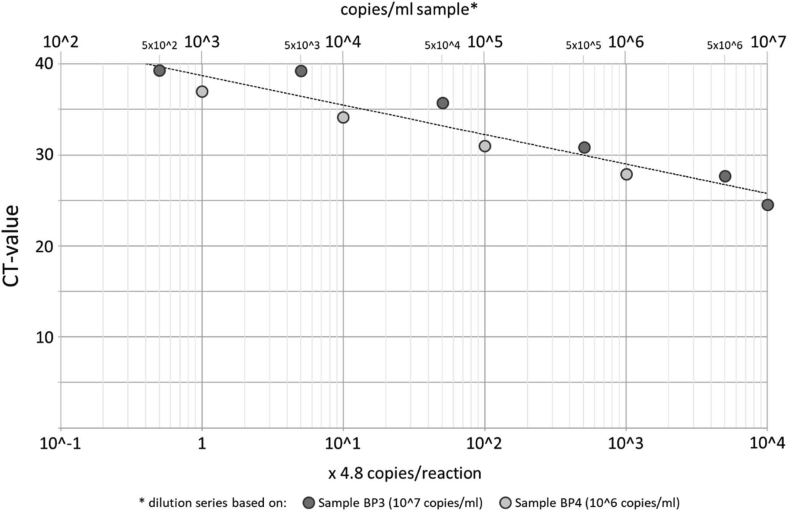
Determination of technical sensitivity. Dilution series of the quantitative reference samples BP3 (10ˆ7 copies/mL) and BP4 (10ˆ6 copies/mL) were performed to correlate Ct-value and viral RNA load.

The next step was to retest throat wash specimens of hospital staff (n = 95), which were previously tested by the Altona Diagnostics RealStar SARS-CoV-2 RT-PCR (Altona Diagnostics, Hamburg, Germany) with the IVD labeled Takara SARS-CoV-2 Direct PCR detection assay. Regarding the Altona PCR 37.9% (n = 36) of the samples were negative and 62.1% (n = 59) were positive for SARS-CoV-2 with Ct-values ranging from 19.89/20.94 (S-/E-gene) to 37.26/37.77 (S-/E-gene) when processed with a COBAS z480 (Roche, Mannheim, Germany). In comparison the overall test sensitivity of the Takara assay was 91.53% (CI: 0.817-0.963) due to deviations regarding samples with low viral load. As no false positive samples were detected the overall specifity was 100% (CI: 0.904-1.0). According to the RKI (Robert Koch-Institut) recommendations [2] Ct-value 30 is set as the critical value to distinguish whether recovered or reconvalescent medical staff is allowed to resume work. For this reason we analysed an additional subcohort (n = 86) including 50 Altona-PCR positive samples with Ct-values < 35. This value was chosen, because analyses regarding the reference samples BP3 and BP4 revealed that N-gene Ct-values are about three cycles later than E-gene Ct-values [1]. The evaluation of the Takara test performance in this Ct-value range resulted in a sensitivity of 98.00% (CI: 0.895–0.997) with still 100% specificity.

While the use of Ct-value thresholds regarding risk-benefit ratio was already discussed [3] the Ct-value 30 is among others considered as a decision-making tool in Germany [2], although it was clearly demonstrated in round robin trials that Ct-values of single specimens may vary seriously among different labs using the same method and even more among different labs using different methods [4].

Leaving aside these issues, the present study show that below Ct-value 35 the Takara assay performs as good as the Altona Real Star SARS-CoV-2 assay. Taking into account the fact that no further RNA-extraction is required, which improves overall processing time, the Takara assay is a real alternative without the need for further equipment. Currently, the company develops the second generation of the assay, which will comply with the European *In Vitro Diagnostics Regulations* (IVDR) guidelines entered into force in Europe in May 2022.

## Transparency declaration

The authors declare no conflicts of interest.
